# Inhibition of AIM2 inflammasome activation alleviates GSDMD-induced pyroptosis in early brain injury after subarachnoid haemorrhage

**DOI:** 10.1038/s41419-020-2248-z

**Published:** 2020-01-30

**Authors:** Bin Yuan, Xiao-ming Zhou, Zong-qi You, Wei-dong Xu, Jie-mei Fan, Shu-juan Chen, Yan-ling Han, Qi Wu, Xin Zhang

**Affiliations:** 1 Department of Neurosurgery, Jinling Hospital, Nanjing Medical University, Nanjing, 210002 P R China; 2 Department of Neurosurgery, Jinling Hospital, Medical School of Nanjing University, Nanjing, 210002 P R China; 3 Department of Neurosurgery, Jinling Hospital, School of Medicine, Jiangsu University, Nanjing, 210002 P R China; 4 Department of Neurosurgery, Jinling Hospital, School of Medicine, Southern Medical University, Nanjing, 210002 P R China

**Keywords:** Cell death in the nervous system, Stroke

## Abstract

Only a few types of inflammasomes have been described in central nervous system cells. Among these, the absent in melanoma 2 (AIM2) inflammasome is primarily found in neurons, is highly specific and can be activated only by double-stranded DNA. Although it has been demonstrated that the AIM2 inflammasome is activated by poly(deoxyadenylic-deoxythymidylic) acid sodium salt and leads to pyroptotic neuronal cell death, the role of AIM2 inflammasome-mediated pyroptosis in early brain injury (EBI) after subarachnoid haemorrhage (SAH) has rarely been studied. Thus, we designed this study to explore the mechanism of gasdermin D(GSDMD)-induced pyroptosis mediated by the AIM2 inflammasome in EBI after SAH. The level of AIM2 from the cerebrospinal fluid (CSF) of patients with SAH was detected. The pathway of AIM2 inflammasome-mediated pyroptosis, the AIM2/Caspase-1/GSDMD pathway, was explored after experimental SAH in vivo and in primary cortical neurons stimulated by oxyhaemoglobin (oxyHb) in vitro. Then, we evaluated GSDMD-induced pyroptosis mediated by the AIM2 inflammasome in AIM2 and caspase-1- deficient mice and primary cortical neurons generated through lentivirus (LV) knockdown. Compared with that of the control samples, the AIM2 level in the CSF of the patients with SAH was significantly increased. Pyroptosis-associated proteins mediated by the AIM2 inflammasome were significantly increased in vivo and in vitro following experimentally induced SAH. After AIM2 and caspase-1 were knocked down by an LV, GSDMD-induced pyroptosis mediated by the AIM2 inflammasome was alleviated in EBI after SAH. Intriguingly, when caspase-1 was knocked down, apoptosis was significantly suppressed via impeding the activation of caspase-3. GSDMD-induced pyroptosis mediated by the AIM2 inflammasome may be involved in EBI following SAH. The inhibition of AIM2 inflammasome activation caused by knocking down AIM2 and caspase-1 alleviates GSDMD-induced pyroptosis in EBI after SAH.

## Introduction

Aneurysmal subarachnoid haemorrhage (SAH) is a life-threatening disease with high mortality and morbidity. Most survivors lose significant quality of life, experiencing long-term physical, neurological and psychological deficits. Blood extravasates into the subarachnoid space, and red blood breakdown products lead to the release of inflammatory cytokines that trigger multiple pathophysiological mechanisms, including vasospasm and tissue injury^1,2^. Evidence shows that early brain injury (EBI) may be responsible for the morbidity and mortality in the early 24–72 h period after SAH^3^.

In recent years, an increased number of studies have indicated that inflammasomes are involved in EBI following SAH. Although many inflammasomes have been identified, only a few have been described and characterised in the central nervous system (CNS)^4^. Absent in melanoma 2 (AIM2), a member of the haemopoietic interferon-inducible nuclear 200 family of proteins, induces the formation of a highly specific type of inflammasome in the neurons that can recognise aberrant double-stranded DNA (dsDNA). AIM2 triggers the formation of inflammasomes that also contain the apoptosis-associated speck-like protein containing a CARD (ASC) and caspase-1 and that induce the cleavage of caspase-1, the maturation of interleukin-1β (IL-1β) and interleukin-18 (IL-18) and pyroptosis. The AIM2 inflammasome mediates pyroptotic neuronal cell death, as has been shown in vivo by incubating cortical neurons with poly(deoxyadenylic-deoxythymidylic) acid sodium salt, a synthetic dsDNA^5^. However, the role of gasdermin D (GSDMD)-induced pyroptosis mediated by the AIM2 inflammasome in the pathogenesis of EBI after SAH has not been clearly elucidated.

GSDMD, a member of the gasdermin protein family, is highly conserved in mammals, but its function has not yet been clarified. Recently, an increasing body of work has suggested that GSDMD is the inducer of pyroptosis^6^. Activated inflammatory caspases efficiently cleave GSDMD at an aspartate site within the linking loop, which enables the release of the GSDMD N-terminus (GSDMD-N), which suspends its auto-inhibition, triggers pyroptosis and binds to phosphatidylinositol phosphates and phosphatidylserine of the cell membrane inner leaflet to induce membrane pore formation and IL-1β secretion^7^. As demonstrated by the crucial role of pyroptosis in immunity and disease, excessive uncontrolled pyroptosis may be detrimental to the host. However, despite these important functions, the potential effects of GSDMD-induced pyroptosis in EBI are still unknown.

Hence, to better understand the mechanism of GSDMD-induced pyroptosis mediated by the AIM2 inflammasome in EBI following SAH, we used an in vivo mouse model of SAH and in vitro cellular model of SAH with oxyhaemoglobin (oxyHb). We also re-assessed GSDMD-induced pyroptosis in EBI following SAH after respectively interfering with the expression of AIM2 and caspase-1 with lentivirus (LV).

## Materials and methods

### Ethics statement

This study was approved by the Ethics Committee of Jinling Hospital and was conducted in accordance with the principles of Good Clinical Practice and the Declaration of Helsinki. All of the patients in this study provided signed informed consent. All animal procedures were approved by the Animal Care and Use Committee of Nanjing Medical University and were performed in accordance with institutional guidelines.

### Clinical sample collection and analysis

Patients with aneurysmal SAH were diagnosed by computerised tomography and digital subtraction angiography. Patients with a history of CNS disease (e.g., CNS infection, stroke, traumatic brain injury, spinal cord injury) or other organ dysfunctions within 6 months were excluded from the study. Cerebrospinal fluid (CSF) samples of SAH patients were collected through a lumbar puncture or ventriculostomy within 72 h after SAH. In contrast, CSF samples from the control group were obtained during spinal anaesthesia before surgery. The samples were collected and immediately centrifuged at 3000 r/min for 10 min at 4 °C and stored at −80 °C until assayed. An equal amount (10 μl) of sample per lane was separated by 12% sodium dodecyl sulfate polyacrylamide gel electrophoresis (SDS-PAGE) and transferred to polyvinylidene-difluoride (PVDF) membrane (MilliporeSigma, Burlington, MA, USA). The membrane was blocked in 5% skimmed milk for 2 h at room temperature and incubated overnight at 4 °C with primary antibodies against AIM2 (1:1000, eBioscience, 14–6008–93) in primary antibody dilution buffer (Beyotime, Nantong, China). After the membrane was washed for 10 min each of three times in TBST, it was incubated with the appropriate HRP-conjugated secondary antibody (1:5000, Bioworld Technology) in secondary antibody dilution buffer (Beyotime, Nantong, China) for 2 h at room temperature. Blotted protein bands were visualised by enhanced chemiluminescence Western blot detection reagents (Amersham, Arlington Heights, IL). Optical densities were obtained using the UN-Scan-It 6.1 software (Silk Scientific, Orem, UT). The team blinded to the clinical parameters of the patients performed the western blot analysis.

### Study design

#### Experiment 1

For in vivo experiments, mice were randomly assigned to various groups: the sham group (*n* = 18), post-SAH 6 h group (*n* = 18), post-SAH 12 h group (*n* = 18), post-SAH 24 h group (*n* = 18), and post-SAH 72 h group (*n* = 18). All mice were sacrificed to collect brain samples for western blot analysis, immunohistochemical staining and Nissl staining.

#### Experiment 2

For in vitro experiments, primary cultured cortical neurons were randomly divided into four groups: the sham group, post-SAH 6 h group, post-SAH 12 h group, post-SAH 24 h group. The neurons were collected for Western blot analysis, flow cytometry analysis, scanning electron microscopy analysis and histopathological study.

#### Experiment 3

For in vivo experiments of AIM2 and caspase-1 knockdown, mice were randomly assigned to five groups: the sham group (*n* = 24), SAH group (*n* = 24), SAH + LV-negative control (NC) group (*n* = 24), SAH + LV-AIM2 group (*n* = 24) and SAH + LV-CAS1 group (*n* = 24). Post-treatment assessments included western blot analysis, immunohistochemical staining, Nissl staining and terminal deoxynucleotidyl transferase-mediated dUTP nick-end labelling (TUNEL) staining.

#### Experiment 4

For in vitro experiments of AIM2 and caspase-1 knockdown, primary cultured cortical neurons were randomly divided into five groups: the sham group, SAH group, SAH + LV-NC group, SAH + LV-AIM2 group, and SAH + LV-CAS1 group. The neurons were collected for western blot analysis, flow cytometry analysis, and TUNEL staining.

### SAH model

In in vivo experiments, the experimental SAH model was performed according to a previous study^8^. Male C57BL/6J mice (8–10 weeks-old, Yangzhou University) were placed under general anaesthesia with 2% isoflurane in 100% O_2_ and maintained with 1% isoflurane. The model was produced by stereotaxic insertion of a needle at 4.5 mm anterior to bregma in the midline at an angle of 40° in the sagittal plane into the prechiasmatic cistern. Concurrently, another mouse was euthanized as a donor for arterial blood by exposed left ventricular cardiac puncture. Nonheparinised arterial blood (60 μl) was withdrawn from a blood donor mouse and slowly injected into the prechiasmatic cistern for 10 s with a 27-gauge needle. The needle was kept in this position for 5 min to prevent backflow or CSF leakage. After the injection, the burr hole was plugged immediately with bone wax. Sham-treated controls received the same procedure with injection of physiologic saline.

In in vitro experiments, oxyHb (MilliporeSigma, Burlington, MA, USA) was prepared and resolved into 20 mM with culture medium and sterilised by filtration through a 0.22-μm sterile filter. As determined in a prior study^9^, neurons were stimulated with 20 mM oxyHb for 24 h to mimic SAH condition.

### Primary neuron culture

Primary cortical neurons were prepared from the cortex of foetal (E16–18) C57BL/6J mice with a modified method as previously described^10^. In brief, the foetal mice were decapitated and placed in 75% alcohol for sterilising. The brain was separated in HBSS (Thermo Fisher Scientific, Waltham, MA, USA) with the aid of a dissection microscope. After the leptomeninges and white matter were carefully removed, the cortex was digested in 0.125% trypsin for 5 min at 37 °C in a water bath. Then, brain tissue suspensions were passed through a 40 μm filter and centrifuged at 1500 r/min for 5 min. The cell pellets were re-suspended in Dulbecco’s modified Eagle’s medium (DMEM, Thermo Fisher Scientific, Waltham, MA, USA) with FBS and penicillin-streptomycin. After that, the suspensions were seeded into poly-d-lysine-coated plates. Two hours later, the culture medium was replaced with Neurobasal Medium (Thermo Fisher Scientific, Waltham, MA, USA) containing GlutaMAX-I (Thermo Fisher Scientific, Waltham, MA, USA), B27 (Thermo Fisher Scientific, Waltham, MA, USA) supplement and penicillin-streptomycin. Cultures were maintained at 37 °C in a humidified atmosphere of 5% CO_2_ and 95% air. Subsequently, half of the medium was replaced every 2 days.

### Lentivirus delivery in vivo and in vitro

AIM2 and caspase-1 protein knockdown were achieved by LV transfection. To establish and maintain the gene knockdown of AIM2 and caspase-1, HBLV-GFP-shAIM2 (LV-AIM2) and HBLV-GFP-shCAS1 (LV-CAS1) were designed and synthesised by Hanbio (Shanghai, China), and HBLV-GFP-PURO (LV-NC) was used as an NC. The viral titre was 1.0 × 10^8^ Tu/ml. The sequences of shRNA were shown as follows: AIM2: CCGGTCCCAGGATTAGTAAACTGAACTCGAGTTCAGTTTACTAATCCTGGGATTTTTG; caspase-1: CCGGCATGGTATCCAGGAGGGAATACTCGAGTATTCCCTCCTGGATACCATGTTTTT; NC: TTCTCCGAACGTGTCACGTAA

In in vivo experiments, mice were anaesthetised and placed in stereotaxic frame. A total of 3 μl of the LV-AIM2, LV-CAS1 or LV-NC was injected into the lateral ventricles at a rate of 0.2 μl /min. The SAH model was established at 72 h after LV injection.

In in vitro experiments, primary cortex neurons were incubated with LV-AIM2, LV-CAS1 or LV-NC according to the manufacturer’s instructions. Our preliminary experiments showed that the multiplicity of infection (MOI) of neurons infected with LV was 30. Neurons were used for subsequent experiments 48 h after initiation of transfection.

### Western blot

The total protein and surface membrane protein samples were extracted from brain tissues of C57BL/6J mice or primary cultured neurons for immunoblotting analysis. For the total protein, the brain sample of mice was removed rapidly after saline perfusion and rinsed in 0.9% normal saline (4 °C) to wash away the blood and blood clot. The samples were lysed in radioimmunoprecipitation assay buffer (RIPA, Beyotime, Nantong, China) containing 1% protease and phosphatase inhibitor cocktails (Roche Inc., Indianapolis, USA). The homogenate after ultrasonic lysis was then centrifuged at 12,000 *×* *g* for 15 min and supernatants (containing cytosolic and membrane fractions) were collected. Extraction of surface membrane protein was conducted using the Membrane and Cytosol Protein Extraction Kit (Beyotime, Nantong, China) according to the manufacturer’s instructions. Protein concentrations were measured with the BCA Kit (Beyotime, Nantong, China). Equal amounts of protein were separated by 10% SDS-PAGE and PVDF membrane. The membrane was blocked with 5% defatted milk for 2 h at room temperature then incubated overnight at 4 °C with primary antibodies against AIM2 (1:1000, eBioscience, 14–6008–93), GSDMD (1:1000, Biorbyt, orb390052, orb593258), GSDMD-N (1:1000, Biorbyt, orb390052, orb593258), ASC (1:1000, Cell Signaling Technology, 67824), caspase-1 (1:1000, Abcam, ab108362; 1:500, MilliporeSigma, ab1871), caspase-1 p20 (1:500, MilliporeSigma, ab1871), caspase-3 (1:1000, Cell Signaling Technology, 9665), cleaved caspase-3 (1:1000, Cell Signaling Technology, 9664), β-actin (1:5000, Bioworld Technology, AP0060), and anti-Na^+^-K^+^ ATPase (1:100000, Abcam, ab76020) in primary antibody dilution buffer (Beyotime, Nantong, China). After the membrane was washed for 10 min each of three times in TBST, the membrane was incubated in the appropriate HRP-conjugated secondary antibody (1:5000, Bioworld Technology) in secondary antibody dilution buffer (Beyotime, Nantong, China) for 2 h at room temperature. The blotted protein bands were visualised by enhanced chemiluminescence (ECL) western blot detection reagents (MilliporeSigma, Burlington, MA, USA) and were exposed to X-ray films.

### Immunofluorescence staining

Immunofluorescence staining was performed as previously described^11^. Briefly, frozen brain sections (7 μm) and cultured cells on coverslips were fixed in ice-cold acetone and 4% paraformaldehyde, respectively. Following treatment with 0.1% Triton X-100, the samples were blocked with 5% FBS before incubation with primary antibody overnight. The samples were washed three times with phosphate buffered saline (PBS) with 0.5% Tween-20 (PBST) for 45 min, incubated with proper secondary antibodies (Alexa Fluor 488, 1:200, Jackson ImmunoResearch Incorporation, West Grove, PA, USA) for 1 h at room temperature, and counterstained with 4,6-diamidino-2-phenylindole (DAPI, 1:2000, MilliporeSigma, Burlington, MA, USA) for 4 min at room temperature. The following antibodies were used: anti-AIM2 (1:100, Bioss, bs-5986R), anti-GSDMD (1:200, Biorbyt, orb390052), anti-neuronal nuclei (NeuN) (1:200, MilliporeSigma, MAB377X), anti-ASC (1:800, Cell Signaling Technology, 67824), anti-caspase-1 p20 (1:50, Santa Cruz Biotechnology, sc-398715), and anti-microtubule associated protein-2 (MAP2) (1:500, MilliporeSigma, MAB3148X).

### TUNEL staining

TUNEL staining was performed according to the manufacturer’s instructions (Roche, South San Francisco, CA, USA). Brain sections or neurons on coverslips were incubated with primary antibody against NeuN at 4 °C overnight. Following washing with PBST, the slides or coverslips were sequentially incubated with TUNEL reaction mixture for 1 h at 37 °C. After washing again, the slides or coverslips were counterstained with DAPI for 4 min. The positive cells were identified, counted, and analysed by two investigators blinded to the grouping.

### Immunohistochemical staining

Mice were euthanised by injection with 5% chloral hydrate in accordance with our approved animal protocol, and the brain tissue was removed. The brain tissues were immediately perfused with 4% paraformaldehyde in PBS after removal and embedded in paraffin for sectioning. Brain sections (4 μm thickness) were incubated overnight at 4 °C with primary antibody against AIM2 (1:1000, eBioscience, 14–6008–93), GSDMD (1:200, Biorbyt, orb390052), ASC (1:50, Santa Cruz Biotechnology, sc-514414), and caspase-1 p20 (1:50, Santa Cruz Biotechnology, sc-398715). After washing carefully in PBS for 15 min, the sections were incubated with HRP-conjugated secondary antibody for 1 h at room temperature. 3,3′-diaminobenzidine (DAB) was used to visualise AIM2, GSDMD, ASC, and caspase-1.

### Enzyme-linked immunosorbent assay

The protein levels of IL-1β and IL-18 secreted in cell culture supernatants were measured using the commercial ELISA kits (eBioscience) according to the manufacturer’s instructions.

### Flow cytometry

Neuron pyroptosis was measured by flow cytometry using propidium iodide (PI) and caspase-1 staining. Briefly, after washing with PBS two times, ~5 × 10^5^ cells were collected by centrifugation (1000 rpm, 5 min). Cells were incubated for 30 min at room temperature with caspase-1 antibody (1:100, Santa Cruz Biotechnology, sc-22163), followed by an appropriate secondary antibody for 30 min at room temperature in the dark. Then, the samples were stained with PI using the Annexin V-FITC Apoptosis Detection Kit (KeyGEN BioTECH, Nanjing, China) according to the protocol of the kit. The assay was carried out using a flow cytometry (Becton-Dickinson, USA). The experiment was repeated at least three times.

### Scanning electron microscopy

Cells were fixed with 2.5% glutaraldehyde overnight and then rinsed three times for 45 min in 0.1 M PBS. The cells were postfixed in 1% osmic acid (OsO_4_) in 0.1 M PBS for 2 h at room temperature followed by dehydration in a graded series of ethanol (30, 50, 70, 80, 90, 95, 100 and 100%) for 15 min at a time and isoamylacetate for 15 min. Samples were dried in Critical Point Dryer. Dried specimens were then sputter coated with gold-palladium and imaged with a scanning electron microscope (HITACHI, SU8100) operating at 3.0 kV.

### Nissl staining

Tissue sections were stained with cresyl violet (Nissl) (Beyotime, Shanghai, China) as described^12^. Nissl staining was performed to evaluate neuronal survival after SAH. Brain sections (4 μm thickness) were hydrated in 1% toluidine blue for 10 min. After washing with double distilled water, they were dehydrated and mounted with Permount. Normal neurons have relatively big cell bodies, rich in cytoplasm, with one or two big round nuclei, whereas damaged cells have shrunken cell bodies, condensed nuclei, dark cytoplasm, and numerous empty vesicles.

### Statistical analysis

All data are presented as the mean ± S.E.M. The expression levels of AIM2 in CSF were compared with the Mann–Whitney U test as follows: between SAH patients and control patients and between the Hunt-Hess grade dichotomies. Statistical analyses between two groups were performed with Student’s t-test and between multiple groups with one-way analysis of variance (ANOVA) followed by the Tukey post hoc test. All statistical analyses were performed using GraphPad Prism 6.0 statistics software (GraphPad Software Inc., San Diego, CA, USA). Statistical significance was inferred at *P* < 0.05.

## Results

### AIM2 in the CSF of patients with SAH

**Fig. 1:**
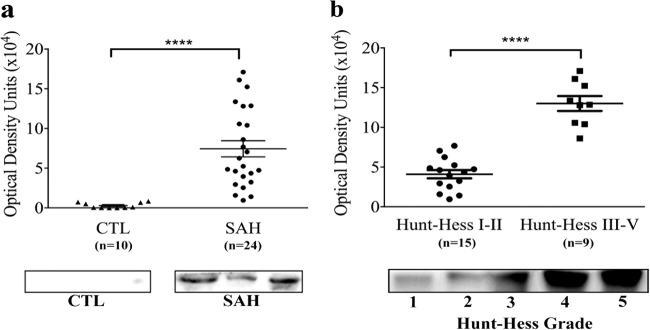
AIM2 levels were measured in CSF from patients. **a** Western blot assay for the AIM2 protein in CSF samples from control and SAH patients; **b** Western blot assay for the AIM2 protein in CSF samples from SAH patients with different Hunt-Hess grade (Control, *n* = 10; SAH, *n* = 24; Hunt-Hess grade I–II, *n* = 15; III–V, *n* = 9). Samples were run on the same gel but were noncontiguous. Data are presented as the mean ± S.E.M. ****P < 0.0001. AIM2, absent in melanoma 2; CSF cerebrospinal fluid; CTL control; SAH subarachnoid haemorrhage.

Compared with the non-SAH control samples, the level of AIM2 was significantly higher in the CSF of patients 1–3 days after they experienced SAH (Fig. 1a). Moreover, the higher the Hunt-Hess grade was, the higher the level of AIM2 found in the CSF (Fig. 1b).

### AIM2 inflammasome-mediated pyroptosis in the temporal cortex

**Fig. 2:**
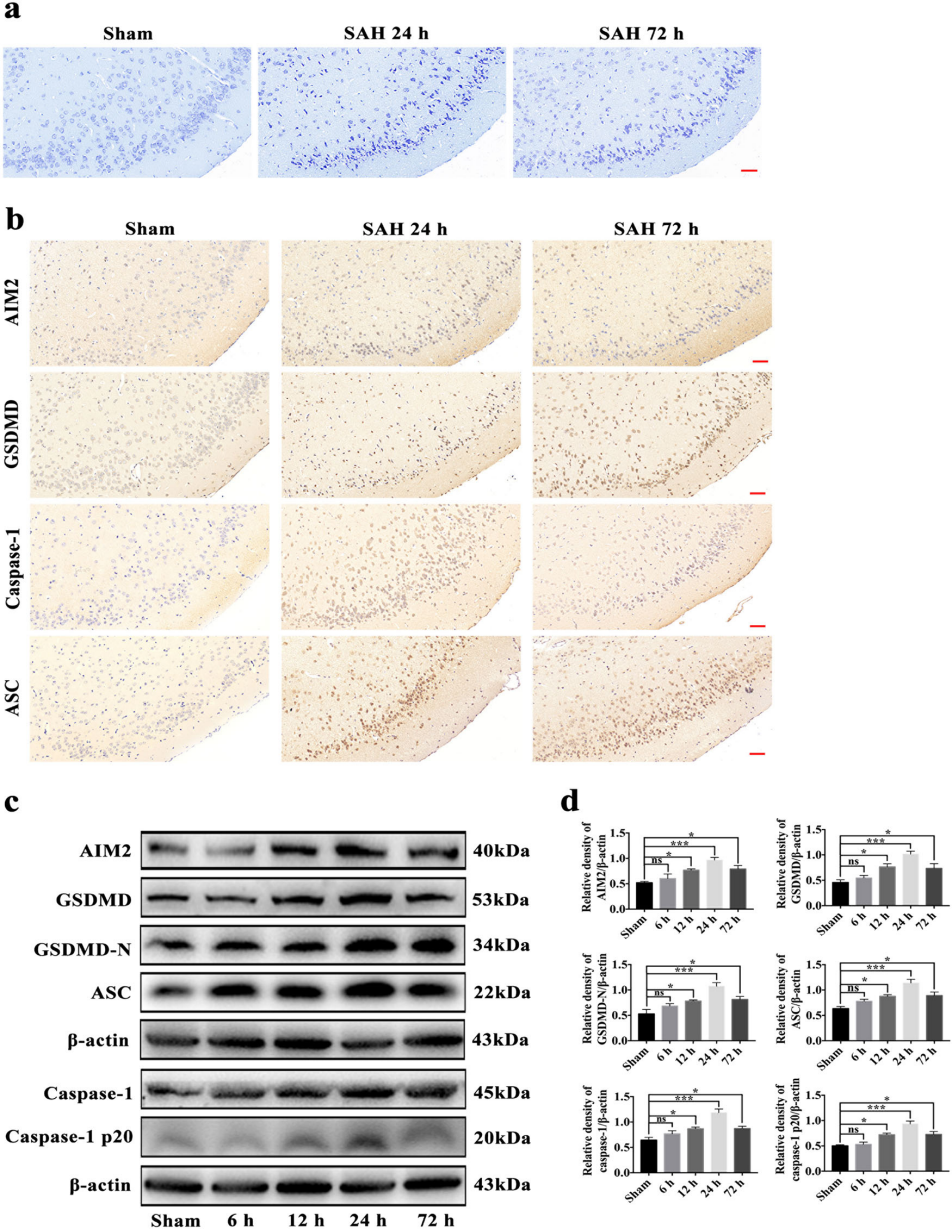
The neuronal AIM2 inflammasome-mediated pyroptosis pathway was explored in the mouse temporal cortex after SAH. **a** Representative Nissl staining images of temporal cortex in sham, post-SAH 24 h and post-SAH 72 h groups; **b** Representative immunohistochemistry images of AIM2, GSDMD, caspase-1 and ASC in sham, post-SAH 24 h and post-SAH 72 h groups; **c** Western blot assay for the expression of AIM2, GSDMD, GSDMD-N, caspase-1, caspase-1 p20 and ASC in temporal cortex after SAH; **d** Quantification of AIM2, GSDMD, GSDMD-N, caspase-1, caspase-1 p20 and ASC. *n* = 6 per group. Bars represent the mean ± S.E.M. *P < 0.05, **P < 0.01, ***P < 0.001, and ns means non-significant. Scale bars = 50 μm. GSDMD gasdermin D; GSDMD-N N-terminus of gasdermin D; ASC apoptosis-associated speck-like protein containing a CARD.

Survival of temporal cortical neurons after SAH was assessed by Nissl staining. The majority of the neurons with large cell bodies and round nuclei were observed in the sham group, while many damaged neurons with shrunken cell bodies, condensed nuclei and dark cytoplasm were observed in the temporal cortex after SAH (Fig. 2a). Immunohistochemical staining showed that AIM2, GSDMD, caspase-1 and ASC were weakly expressed in the sham group, while the expression of AIM2, GSDMD, caspase-1 and ASC was evidently increased in the temporal cortex at 24 and 72 h after SAH (Fig. 2b). Western blotting was performed to assess the expression of AIM2, GSDMD, GSDMD-N, caspase-1, caspase-1 p20 and ASC after SAH in different time course experiments. As shown (Fig. 2c, d), the expression level of AIM2, GSDMD, GSDMD-N, caspase-1, caspase-1 p20 and ASC was low in the sham group, while it was significantly increased, peaking at 24 h and alleviating at 72 h after SAH in the experimental groups. There was a statistically significant difference between the levels in the sham group and those in the 24- and 72-h groups.

### GSDMD-induced pyroptosis mediated by the AIM2 inflammasome in primary cortical neurons

**Fig. 3:**
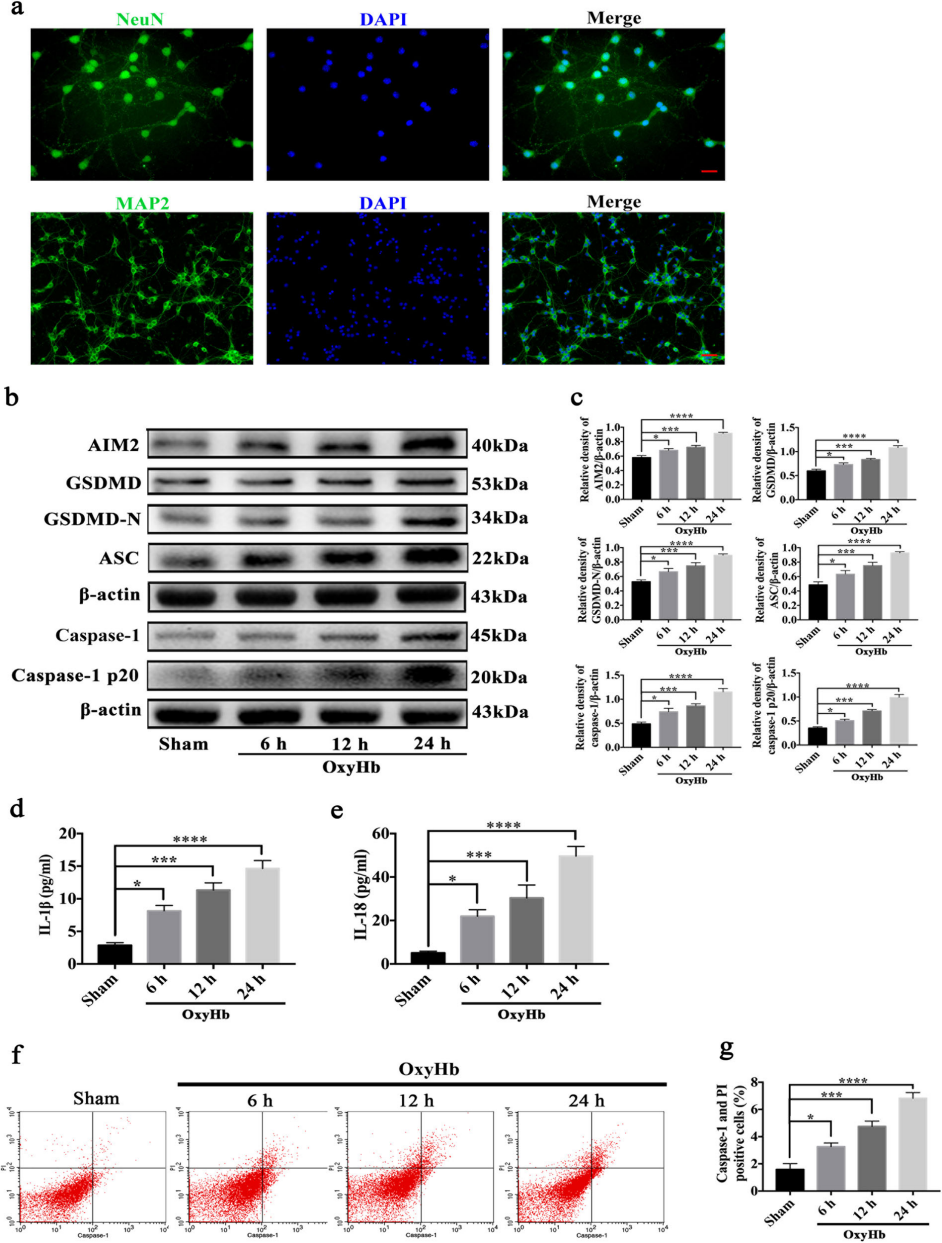
The AIM2 inflammasome-mediated pyroptosis pathway was measured in primary cortical neurons exposed to oxyHb. **a** Representative images of cultured primary cortical neurons, scale bars (NeuN) = 50 μm, scale bars (MAP2) = 200 μm; **b** Western blot assay for the expression of AIM2, GSDMD, GSDMD-N, caspase-1, caspase-1 p20 and ASC in all groups; **c** Quantification of AIM2, GSDMD, GSDMD-N, caspase-1, caspase-1 p20 and ASC; **d**, **e** Quantitative analysis of IL-1β and IL-18 secreted in the supernatant; **f** Flow cytometry analysis of neurons in all groups; **g** Quantification of caspase-1 and PI-positive neurons in all groups. Bars represent the mean ± S.E.M. *P < 0.05, ***P < 0.001, ****P < 0.0001 and ns means non-significant. OxyHb oxyhaemoglobin; NeuN neuronal nuclei; MAP2 microtubule associated protein 2; IL-1β interleukin-1β; IL-18 interleukin-18; PI propidium iodide.

**Fig. 4:**
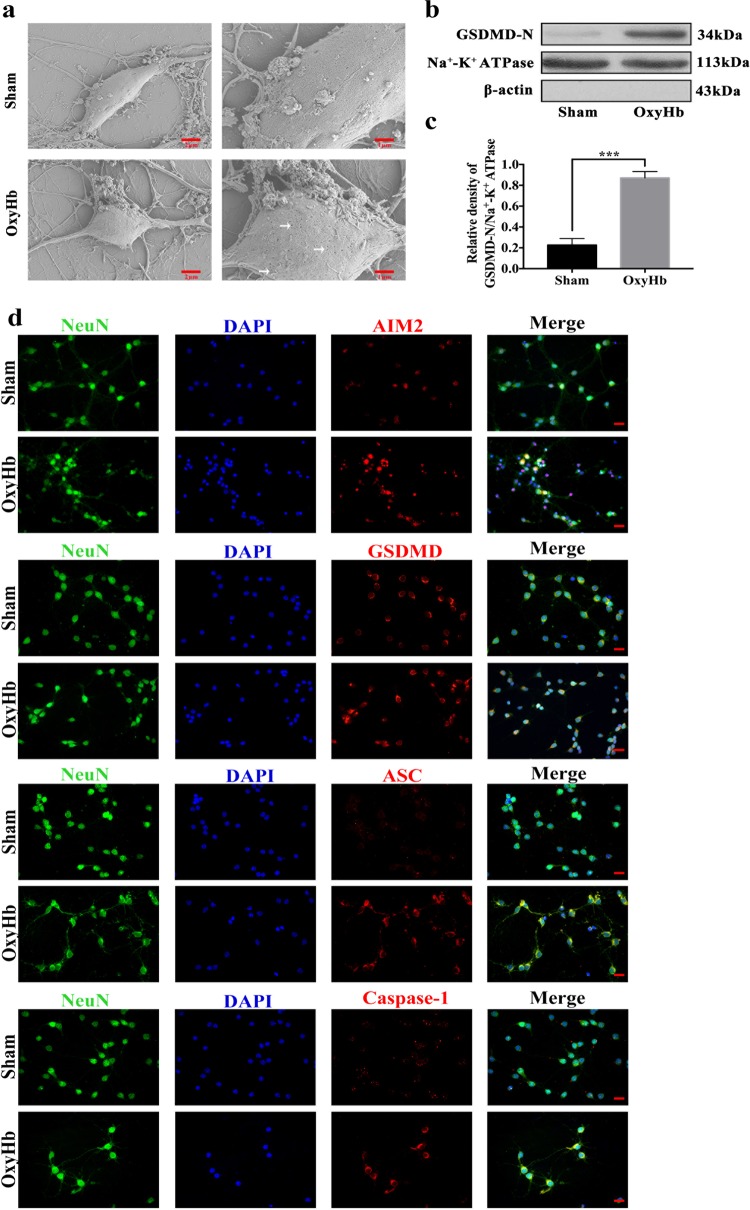
Pyroptosis in primary cortical neurons. **a** Representative scanning electron microscopy images in sham and oxyHb groups; **b** Western blot assay for the expression of GSDMD-N in the cell membranes; **c** Quantification of GSDMD-N; **d** Representative immunofluorescence staining images of AIM2, GSDMD, caspase-1 and ASC in control and oxyHb-exposed 24 h groups. Scale bars = 50 μm. White arrows show the pores caused by the N-terminal fragment of GSDMD. Bars represent the mean ± S.E.M. ***P < 0.001.

To investigate the role of GSDMD-induced pyroptosis mediated by the AIM2 inflammasome in neurons after SAH, we isolated and cultured mouse cortical neurons (Fig. 3a). These primary cortical neurons were stimulated by oxyHb, which simulates SAH in vivo. The results from the Western blot analysis showed that the expression levels of AIM2, GSDMD, GSDMD-N, caspase-1, caspase-1 p20 and ASC were significantly increased in primary cortical neurons exposed to oxyHb compared with the levels in the sham group (Fig. 3b, c). Moreover, as the time of oxyHb stimulation was prolonged, the expression of the pyroptosis-related proteins mediated by the AIM2 inflammasome continuously increased. Then, double immunofluorescence staining was performed to identify where these proteins were mainly expressed in the neurons exposed to oxyHb. As shown in Fig. 4d, in the sham group, AIM2, GSDMD, caspase-1 and ASC were weakly expressed in the neurons. However, the expression levels of the pyroptosis-related proteins were enhanced after the neurons were stimulated by oxyHb compared with their expression levels in the sham group. Double immunofluorescence staining also revealed that AIM2 was mainly expressed in the nucleus of the cultured primary neurons in the sham group, while it was expressed in both the nucleus and cytoplasm after SAH. In contrast, GSDMD, caspase-1 and ASC were mainly expressed in the cytoplasm.

The secretion of the synthesised pro-inflammatory mediators IL-18 and IL-1β plays an important role in EBI; thus, we detected the concentrations of IL-18 and IL-1β in the medium supernatant using an ELISA kit. As shown in Fig. 3d, e, compared to the low levels in the sham group, the levels of IL-18 and IL-1β were significantly increased after the neurons were stimulated by oxyHb.

Flow cytometry with PI and caspase-1 staining was used to detect pyroptosis induced by GSDMD (Fig. 3f, g). The results revealed that pyroptosis had obviously occurred in the primary cortical neurons exposed to oxyHb.

### Pyroptosis in neurons

One of the morphological features of pyroptosis is that the activated GSDMD-N oligomerizes at the pores of the cell membrane. To observe the changes in the neuronal cell membrane after the neurons were stimulated by oxyHb, scanning electron microscopy was used. As shown in Fig. 4a, a large number of pores were formed on the neuronal cell membrane after the neurons were stimulated by oxyHb, while few pores were observed in the cells of the sham group. Furthermore, western blot analysis of the membrane proteins showed that the activated GSDMD-N aggregated at the cell membrane (Fig. 4b, c). Thus, it was observed that pyroptosis is involved in neuronal injury after SAH.

### Effects of inhibited AIM2 inflammasome activation on GSDMD-induced pyroptosis in vivo

**Fig. 5:**
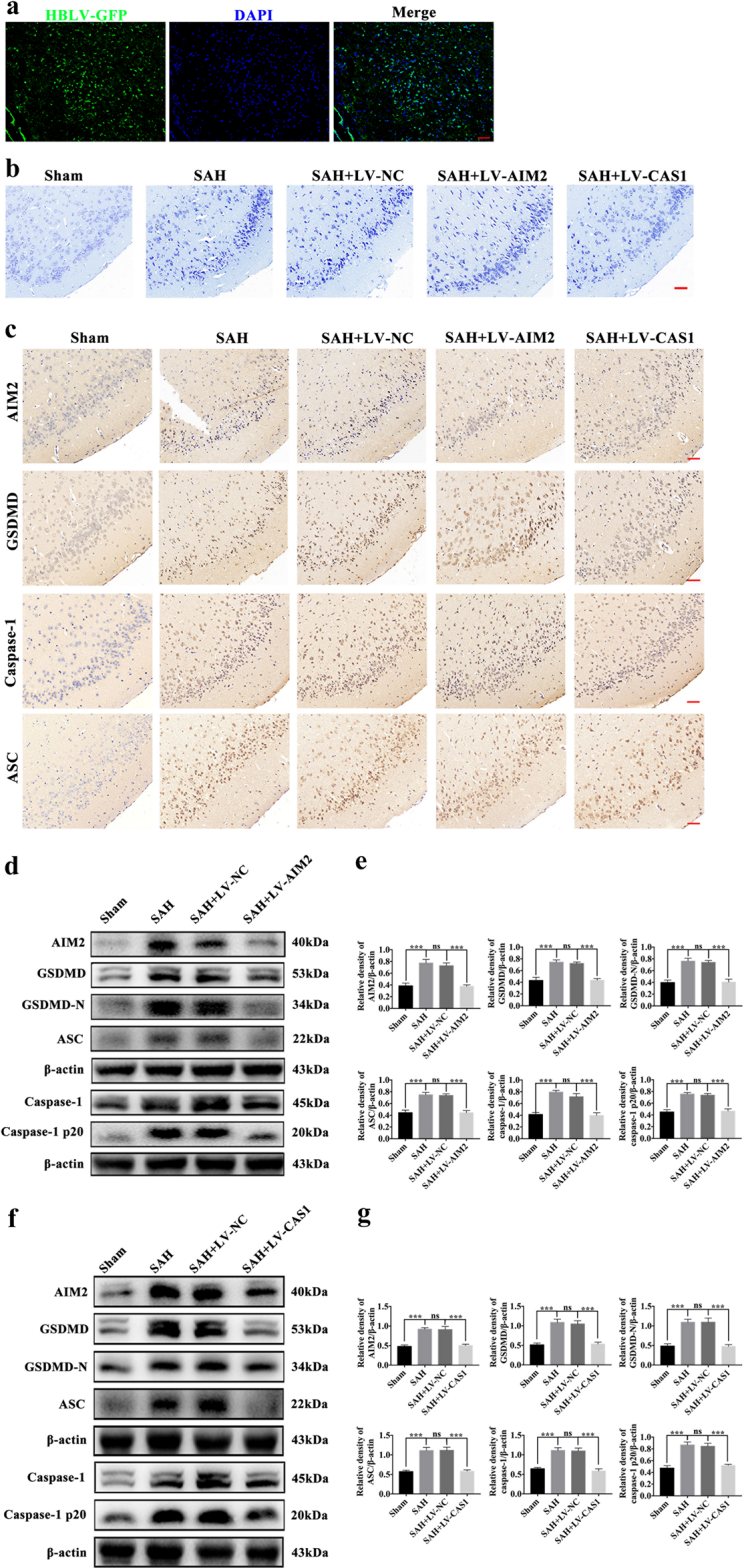
Effects of inhibited AIM2 inflammasome activation on GSDMD-induced pyroptosis 24 h post-SAH in vivo. **a** The transfection of LV-NCGFP in vivo, scale bars = 200 μm; **b** Representative immunohistochemistry images of AIM2, GSDMD, caspase-1 and ASC in all groups, scale bars = 50 μm; **c** Representative Nissl staining images of temporal cortex in all groups, scale bars = 50 μm; **d**, **f** Western blot assay for the expression of AIM2, GSDMD, GSDMD-N, caspase-1, caspase-1 p20 and ASC in all groups; **e**, **g** Quantification of AIM2, GSDMD, GSDMD-N, caspase-1, caspase-1 p20 and ASC. n = 6 per group. Bars represent the mean ± S.E.M. ***P < 0.001, and ns means non-significant. DAPI 4,6-diamidino-2-phenylindole.

To determine the effects of AIM2 inflammasome activation in GSDMD-induced neuronal pyroptosis after SAH, we evaluated the expression of pyroptosis-related proteins mediated by the AIM2 inflammasome by knocking down AIM2 and caspase-1 after SAH. Immunofluorescence staining showed that LV was transfected with high efficiency into the temporal cortex (Fig. 5a). In addition, Nissl staining revealed that, although the temporal cortical neurons were dramatically damaged by SAH, the damage was reversed when AIM2 and caspase-1 were knocked down (Fig. 5b). Further immunohistochemical staining also showed that pyroptosis of the temporal cortical neurons was significantly inhibited by the high transfection efficiency of the LV-AIM2 and LV-CAS1 (Fig. 5c). The results from the Western blot analysis suggested that, compared with the levels in the SAH and SAH + LV-NC groups, the expression levels of AIM2, GSDMD, GSDMD-N, caspase-1, caspase-1 p20 and ASC were significantly decreased in the SAH + LV-AIM2 group. However, there was no difference in the expression levels of these pyroptosis-related proteins between the SAH and SAH + LV-NC groups (Fig. 5d, e). When caspase-1 was knocked down, Western blot analysis also showed that the expression levels of pyroptosis-related proteins mediated by the AIM2 inflammasome dramatically decreased in the SAH + LV-CAS1 group compared with those in the SAH + LV-NC group (Fig. 5f, g).

### Effects of inhibited AIM2 inflammasome activation on GSDMD-induced pyroptosis in vitro

**Fig. 6:**
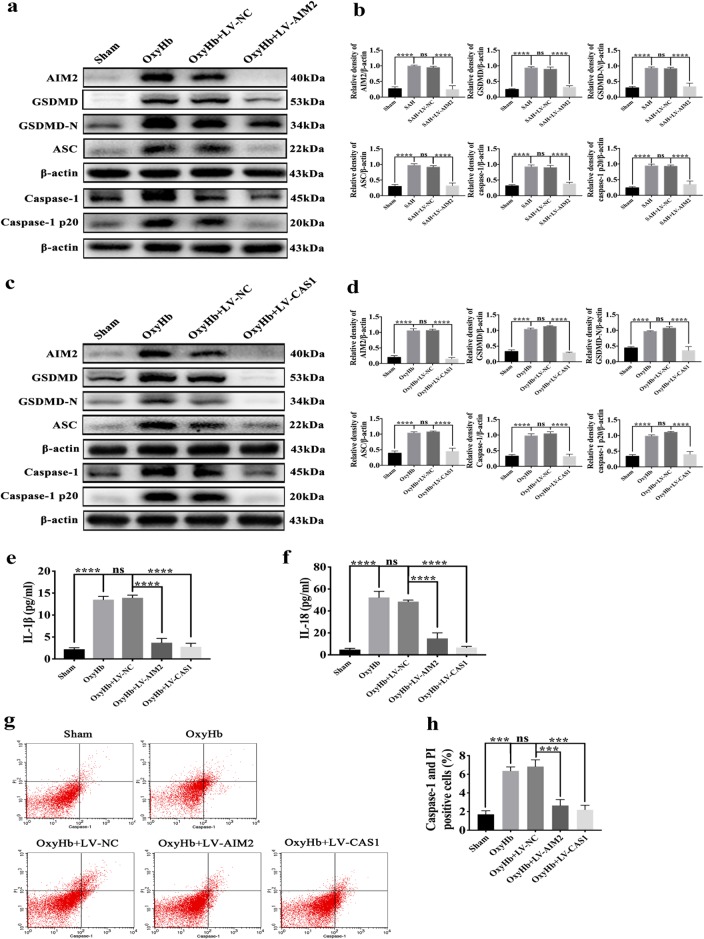
Effects of inhibited AIM2 inflammasome activation on GSDMD-induced pyroptosis in primary cortical neurons exposed to oxyHb. **a**, **c** Western blot assay for the expression of AIM2, GSDMD, GSDMD-N, caspase-1, caspase-1 p20 and ASC in all groups; **b**, **d** Quantification of AIM2, GSDMD, GSDMD-N, caspase-1, caspase-1 p20 and ASC; **e**, **f** Quantitative analysis of IL-1β and IL-18 secreted in the supernatant; **g** Flow cytometry analysis of neurons in all groups; **h** Quantification of Caspase-1 and PI-positive neurons in all groups. Bars represent the mean ± S.E.M. ***P < 0.001, ****P < 0.0001 and ns means non-significant.

Cultured primary cortical neurons were also transfected with LV-AIM2, LV-CAS1 or LV-NC. The expression levels of AIM2, GSDMD, GSDMD-N, caspase-1, caspase-1, p20 and ASC were also dramatically decreased by knocking down AIM2 in the OxyHb + LV-AIM2 group compared with the OxyHb and OxyHb + LV-NC groups (Fig. 6a, b). When caspase-1 was knocked down, the expression of pyroptosis-related proteins was also significantly reduced (Fig. 6c, d). After knocking down AIM2 and caspase-1, the amount of IL-18 and IL-1β secretion obviously decreased (Fig. 6e, f). Moreover, the results from flow cytometry showed that GSDMD-induced pyroptosis mediated by the AIM2 inflammasome was significantly alleviated after the AIM2 or caspase-1-deficient neurons were exposed to oxyHb (Fig. 6g, h).

### Effects on apoptosis after knocking down caspase-1 by an LV following SAH

**Fig. 7:**
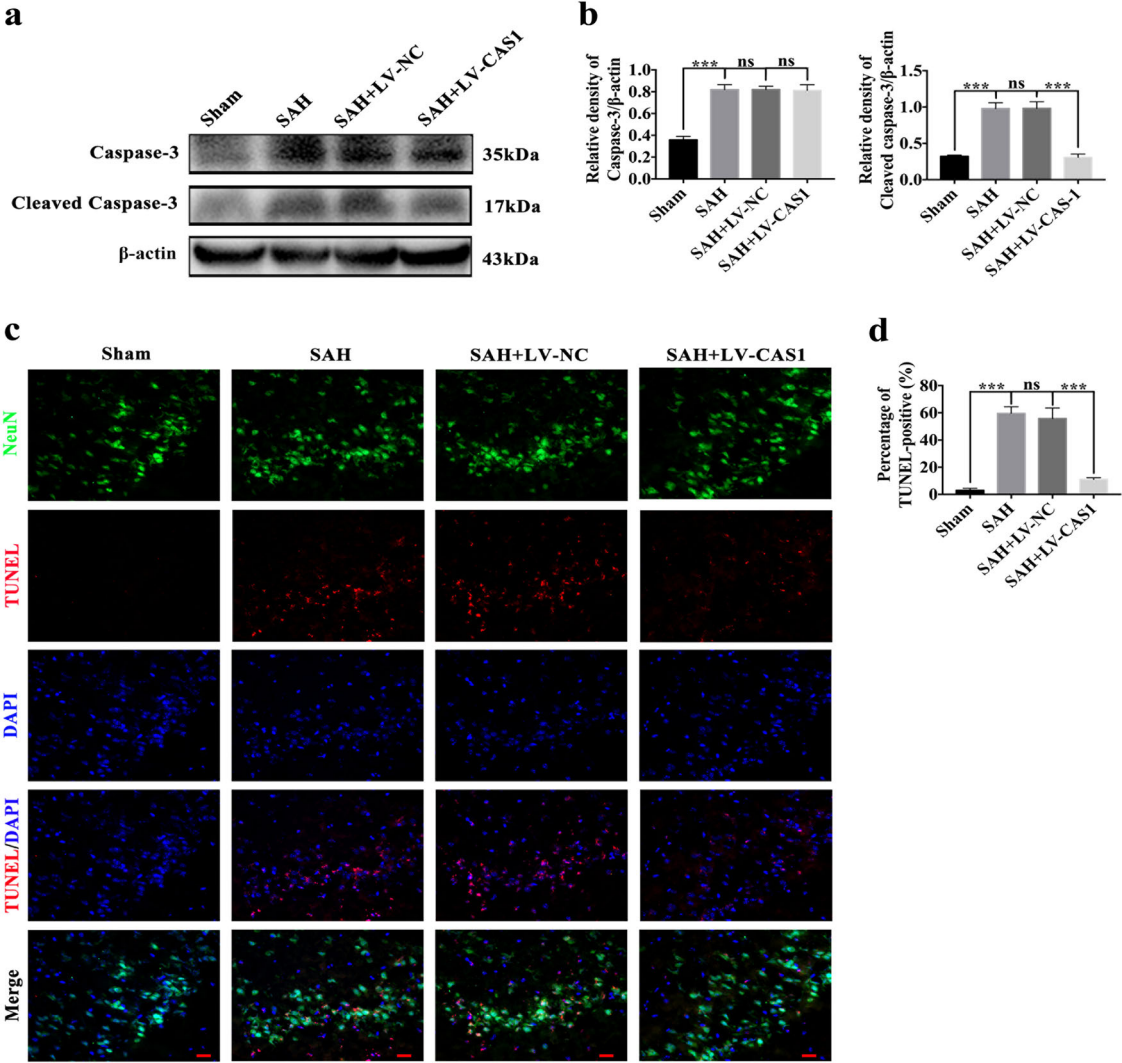
Effects of inhibited GSDMD activation by knocking down caspase-1 on apoptosis 24 h post-SAH in vivo. **a** Western blot assay for the expression of caspase-3 and cleaved caspase-3 in all groups; **b** Quantification of caspase-3 and cleaved caspase-3; **c** Representative immunofluorescence images of TUNEL staining; **d** Quantification of TUNEL-positive neurons. *n* = 6 per group. Bars represent the mean ± S.E.M. ***P < 0.001 and ns means non-significant. Scale bars = 50 μm.

**Fig. 8:**
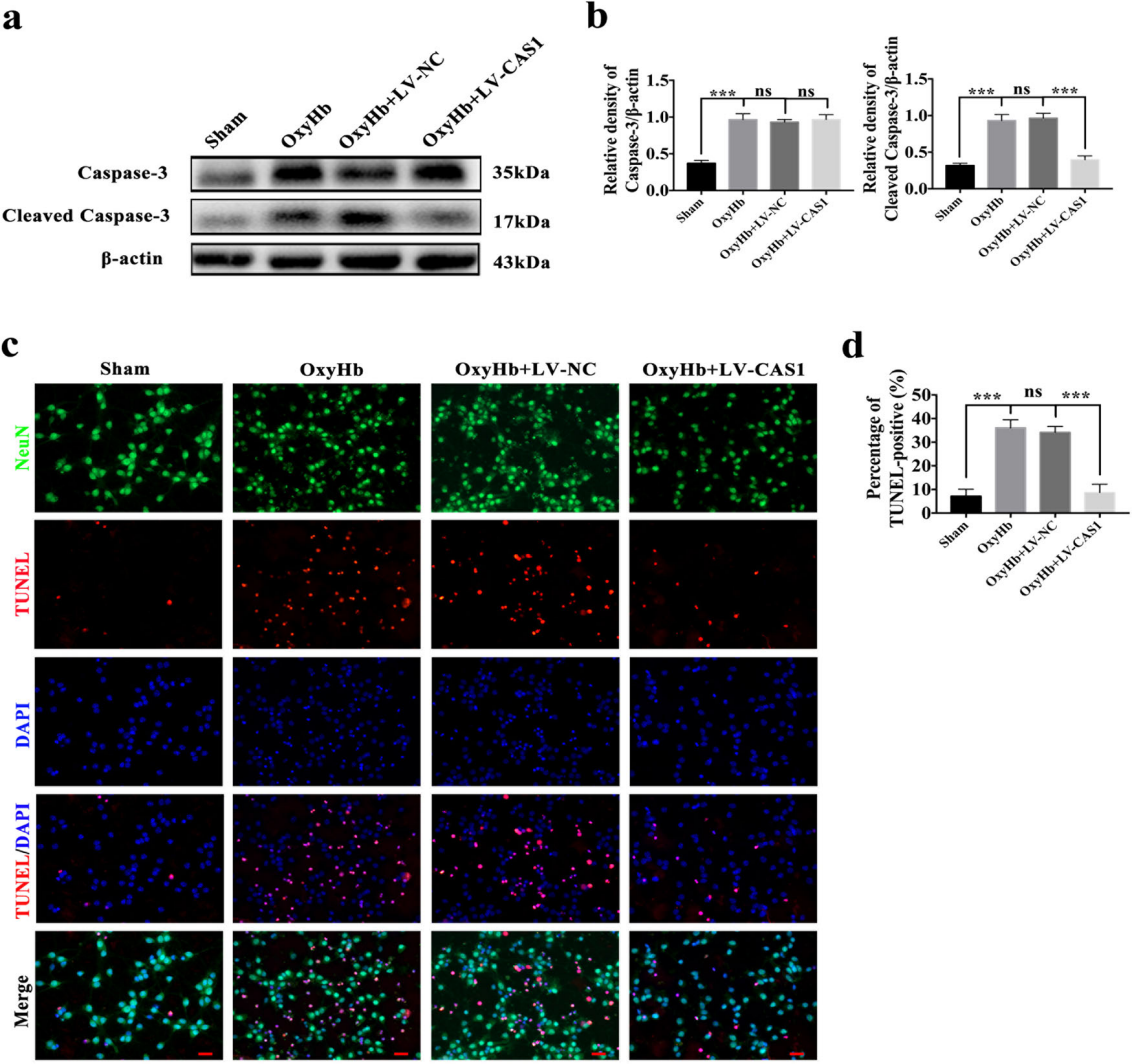
Effects of inhibited GSDMD activation by knocking down caspase-1 on apoptosis in primary cortical neurons exposed to oxyHb. **a** Western blot assay for the expression of caspase-3 and cleaved caspase-3 in all groups; **b** Quantification of caspase-3 and cleaved caspase-3; **c** Representative immunofluorescence images of TUNEL staining; **d** Quantification of TUNEL-positive neurons. Bars represent the mean ± S.E.M. ***P < 0.001 and ns means non-significant. Scale Bars = 50 μm.

To determine the effect of the inhibition of caspase-1 activation after SAH on apoptosis, we explored the levels of caspase-3 expression in vivo and in vitro. As shown in Figs. 7a, b and 8a, b, there was no significant difference in the expression of caspase-3 between the SAH + LV-NC and SAH + LV-CAS1 groups. However, compared with the level in the SAH + LV-NC group, the level of activated caspase-3 decreased significantly in the SAH + LV-CAS1 group. TUNEL staining also suggested that the number of TUNEL-positive neurons were dramatically reduced after SAH by caspase-1 knockdown (Figs. 7c, d and 8c, d). Thus, knocking down caspase-1 maybe affected not only GSDMD activation-induced pyroptosis but also caspase-3 activation-induced apoptosis.

## Discussion

In the present study, we studied the possible role of GSDMD-induced pyroptosis mediated by the AIM2 inflammasome in the pathogenesis of EBI after SAH. The main findings can be summarised as follows: (1) Pyroptosis-related proteins mediated by the AIM2 inflammasome were upregulated in the brain temporal cortex after SAH and in primary cortical neurons exposed to oxyHb. The expression levels of AIM2, GSDMD, GSDMD-N, caspase-1, caspase-1 p20 and ASC increased continuously in a time-dependent manner. (2) The results from the immunohistochemistry and immunofluorescence staining showed that AIM2 inflammasome-mediated pyroptosis mainly occurred in brain neurons. (3) The inhibition of AIM2 inflammasome activation by knocking down AIM2 and caspase-1 could suppress GSDMD-induced neuronal pyroptosis by decreasing the expression and activation of the pyroptosis-related proteins that are mediated by the AIM2 inflammasome. (4) Knocking down caspase-1 suppressed not only GSDMD activation-induced neuronal pyroptosis but also caspase-3 activation-induced neuronal apoptosis. These findings suggested, for the first time, that pyroptosis could be involved in EBI after SAH through the AIM2/Caspase-1/GSDMD pathway. Moreover, inhibiting the expression and activation of caspase-1 could alleviate both pyroptosis and apoptosis (Supplementary Figure).

The pathophysiology of SAH is complicated and involves multiple pathogenic mechanisms (e.g., inflammation, oxidative stress, and apoptosis). A growing body of evidence indicates that the inflammatory response plays a vital role in injury expansion and brain damage after SAH, including EBI, vasospasm, and delayed neurological deterioration^2^. In recent years, inflammasomes have been demonstrated to be important participants in EBI after SAH^10^. The inflammasome is a cytosolic multimeric signalling complex that responds to invading pathogens and host-derived danger signals such that its activation leads to caspase-1 activation. Inflammasome activation proceeds via the formation of a multimolecular complex containing a receptor, an adaptor such as ASC and the cysteine protease caspase-1^13^. Then, activated caspase-1 triggers the maturation of the pro-inflammatory cytokines IL-1β and IL-18 and induces pyroptotic cell death. Several nucleotide-binding oligomerisation domain (NOD)-like receptors (NLRs), as well as AIM2-like receptors (ALRs), have been shown to form inflammasomes. Most of the inflammasomes described to date contain an NLR protein, namely, NOD-like receptor containing pyrin domain 1(NLRP1), NLRP2, NLRP3, NLRP6, NLRP7, NLRP12, and NLR- and caspase-activating recruitment domain-containing 4 (NLRC4). However, only a few inflammasomes have been studied in the CNS, namely, NLRP1, NLRP2, NLRP3 and AIM2. Among these inflammasomes, the NLRP3 inflammasome has been the most studied. NLRP3 inflammasome-mediated neuroinflammation is involved in many acute and chronic CNS diseases, including SAH^10,14,15^. In SAH, activation of the NLRP3 inflammasome is also essential for the modulation of pro-inflammatory cytokines, and inhibition of the NLRP3 inflammasome by pharmacological treatment can alleviate brain injury after SAH^10,16^.

In addition to NLRP3, AIM2 is also an important inflammasome involved in CNS infection and injury^17^. In the present study, we collected and analysed CSF samples from patients with or without SAH. The results from the analysis of patient-derived CSF samples revealed that the level of the AIM2 protein in the CSF of the SAH patients was significantly greater than that in the CSF from non-SAH patients. The higher the Hunt-Hess grade was, the higher the level of AIM2 that was found in the CSF. In the experimental SAH model, we also found that the AIM2 inflammasome was activated in vivo at 24 h post-SAH and that in vitro, the AIM2 inflammasome was significantly unregulated in cultured cortical neurons 6 h after incubation with oxyHb and the levels continued to increase for 12 and 72 h. To verify the role of GSDMD-induced pyroptosis mediated by the AIM2 inflammasome in EBI after SAH, LV was used to knock down the expression of AIM2. The results showed that AIM2 inflammasome-mediated pyroptosis was significantly alleviated in EBI following SAH.

As reported, the AIM2 inflammasome could be activated by aberrant dsDNA, including bacterial DNA and viral DNA, from pathogens and hosts and by endogenous self-DNA within the cytosol, damaged DNA within the nucleus, and self-DNA secreted by exosomes^18^. Wang et al. detected CSF DNA levels in patients with SAH and found that both nuclear and mitochondrial DNA levels in the CSF were significantly increased in the patients with SAH compared with volunteers, and the CSF nuclear and mitochondrial DNA levels were significantly higher on days 1 and 4^19^. Moreover, higher CSF DNA levels were associated with worse outcomes for patients with SAH^19^. This result was consistent with the increased AIM2 in the SAH patients in our study. Combined with the results of this study showing that the level of AIM2 in CSF increased significantly within 3 days after SAH, we found that the elevated level of DNA in the CSF after SAH was synchronous and consistent with that of AIM2. Thus, we speculate that pyroptosis mediated by the AIM2 inflammasome is involved in EBI after SAH. This kind of EBI should be considered, to be exact, a secondary brain injury.

Recently, some scholars believe that pyroptosis should be redefined as GSDMD-mediated, rather than caspase-1-mediated, programmed necrosis^20^. GSDMD was discovered to form a pore and act as an effector for pyroptosis. In GSDMD-deficient cells, pyroptosis cannot be triggered by known canonical inflammasome ligands^21^. GSDMD contains ~480 amino acids in two domains, and the N-terminal gasdermin domain (GSDMD-N) and the C-terminal gasdermin domain (GSDMD-C) are linked by a long loop. Mounting evidence has demonstrated that GSDMD plays a key role in CNS injury^22,23^. In our study, we also observed that GSDMD was upregulated after SAH. Although GSDMD is considered the initiator of pyroptosis, GSDMD-N is the direct and sole effector of pyroptosis. Activated caspase-1 or caspase-11 efficiently cleaves GSDMD at a conserved glutamic acid residue (D276 in mouse and D275 in humans)^21,24^. The cleaved GSDMD unleashes the pro-pyroptotic N-terminal fragment from the auto-inhibited state maintained by the C-terminus, thus separating the role of GSDMD into that of GSDMD-N and that of GSDMD-C. The released GSDMD-N is oligomerized, and only the oligomerised form of GSDMD-N is able to translocate to the plasma membrane, where it induces cell rupture and the release of inflammatory cytokines, such as IL-1β^25^. We also observed that the GSDMD-N generated by caspase-1 cleavage forms an oligomer and migrates to the plasma membrane to kill cells.

Furthermore, GSDMD-N alone localises to the plasma membrane, as evidenced by GSDMD-N binding to liposomes in studies in vitro. Binding studies with liposomes and lipid strips revealed that GSDMD-N has a high affinity for liposomes containing lipids such as cardiolipin, phosphatidylinositol 4-phosphate [PI(4)P] and phosphatidylinositol 4,5-bisphosphate [PI(4,5)P] compared with other lipid types^26^. It is through its lipid-binding specificity that GSDMD-N disrupts plasma membranes, which it perpetrates only when exposed to the cytosolic PI-containing inner leaflet but not when exposed to the extracellular outer leaflet, which lacks PI^26^. While GSDMD-N kills from within the cell, its released form does not harm neighbouring cells. Mounting evidence has shown that the inner ring diameter of the pore formed by GSDMD-N is estimated to be between 10 and 20 nm^6,27,28^. Different methods have revealed a pore consisting of 16 or 24 GSDMD-N units^27,28^. This finding is consistent with the observation we made in the present study revealing that a large number of pore-like structures appear on the surface of neurons stimulated by oxyHb. The results of the membrane protein analysis suggest that the pore-like structure may be caused by the oligomerization of GSDMD-N at the cell membrane.

In the present study, we also interfered with the expression of caspase-1 by an LV. After caspase-1 was knocked down, GSDMD-induced pyroptosis was markedly reduced in EBI following SAH. Knocking down caspase-1 inhibited not only the expression and activation of GSDMD but also the expression of pyroptosis-related proteins mediated by the AIM2 inflammasome. We propose two explanations for this result: First, caspase-1-mediated inflammatory injury could be greatly alleviated after caspase-1 is knocked down. Caspase-1 is unequivocally required for the proteolytic processing of IL-1β and IL-18; these two cytokines engage their transmembrane receptors IL-1R and IL-18R to promote inflammation via the activation of nuclear factor-kappaB transcriptional programmes that are induced by the activation of myeloid differential protein-88, a key signalling adaptor^29^. When caspase-1 was knocked down, caspase-1-mediated inflammatory injury may have been alleviated, and the amount of dsDNA released from damaged cells could be reduced. Second, the non-functional AIM2/ASC complex was quickly depleted. Juruj et al. found that a negative feedback loop controlled by ASC/caspase-1 regulates AIM2 complex formation/stability^13^. In the absence of a functional AIM2 inflammasome, the AIM2/ASC complex formed very rapidly. However, this complex was then removed through activated autophagy following AIM2 speck formation^13^. Thus, when caspase-1 levels were deficient, the AIM2 inflammasome was not readily formed and pyroptosis was thus mediated after SAH.

Intriguingly, knocking down caspase-1 alleviated not only GSDMD activation-induced neuronal pyroptosis but also caspase-3 activation-induced neuronal apoptosis. A few other studies have reported that GSDMD-deficient cells may die because of the caspase-1 cleavage of caspase-3/7^21,30^. Inhibiting caspase-1 activity may have attenuated caspase-3-dependent apoptosis, but the mechanism remains unclear^31–33^. We found that caspase-1 was essential for the activation of caspase-3. The cleavage of caspase-3 was significantly hindered when caspase-1 was knocked down in EBI following SAH. However, the expression of caspase-3 was not affected by caspase-1 knockdown. Therefore, we speculated that apoptosis was also alleviated in EBI after SAH, because knocking down caspase-1 hindered the cleavage of caspase-3. It was recently reported that GSDMD was also cleaved during apoptosis. GSDMD was cleaved by caspase-3/7 at D87 during apoptosis, while GSDMD was cleaved by caspase-1 at D275/D276 during pyroptosis^34^. The p30 N-terminal fragment of GSDMD (GSDMD p30) released by the cleavage at D275/D276 forms pores in the plasma membrane, thereby mediating pyroptotic cell death. Nevertheless, this cleaved GSDMD also generated the p43 fragment of GSDMD (GSDMD p43) at D87 during apoptosis, which inactivated or failed to trigger pyroptosis (Supplementary Figure). We preliminarily found that caspase-1 may be an important protein at the intersection of the pyroptosis and the apoptosis pathways. Inhibition of caspase-1 activity alleviated not only GSDMD activation-induced neuronal pyroptosis but also caspase-3 activation-induced neuronal apoptosis.

In this study, we demonstrated, for the first time, that the GSDMD-induced pyroptosis mediated by the AIM2 inflammasome may be involved in EBI after SAH. When inhibiting the activation of the AIM2 inflammasome by knocking down AIM2 and caspase-1 with LV, the pyroptosis mediated by the AIM2 inflammasome was significantly alleviated. The activation of caspase-3 was also decreased after caspase-1 was knocked down. However, a clearer regulatory mechanism between pyroptosis and apoptosis remains to be further explored.

## Supplementary information

Supplementary table

Supplementary figure
