# Supplementary material for: Inhibition of AIM2 inflammasome activation alleviates GSDMD-induced pyroptosis in early brain injury after subarachnoid haemorrhage
Source: Cell Death Dis. 2020 Jan 30;11(1):76. doi: 10.1038/s41419-020-2248-z (PMC6992766; doi:10.1038/s41419-020-2248-z)
Supplement: Supplementary file 1 — Supplementary table [file 41419_2020_2248_MOESM1_ESM.docx]

**Table：Clinical data from SAH cases**

| Case No. | Sex | Age | Aneurysm Location | Hunt-Hess Grade |
| --- | --- | --- | --- | --- |
| 1 | M | 50 | ACA | II |
| 2 | M | 41 | PcoA | I |
| 3 | M | 61 | AcoA | II |
| 4 | F | 42 | AcoA | II |
| 5 | F | 52 | PcoA | II |
| 6 | F | 47 | PcoA | I |
| 7 | F | 59 | PcoA | I |
| 8 | M | 47 | AcoA | I |
| 9 | F | 65 | ACA, MCA | II |
| 10 | F | 52 | PcoA | II |
| 11 | F | 52 | PICA | II |
| 12 | M | 51 | AcoA | II |
| 13 | F | 36 | MCA | I |
| 14 | M | 50 | ACA | II |
| 15 | M | 69 | PcoA | II |
| 16 | M | 41 | AcoA | III |
| 17 | F | 59 | PICA | III |
| 18 | F | 42 | PcoA | III |
| 19 | F | 57 | AcoA | IV |
| 20 | F | 59 | PcoA, MCA | III |
| 21 | M | 49 | VADA | IV |
| 22 | F | 69 | AcoA, MCA | IV |
| 23 | M | 49 | AcoA, MCA | IV |
| 24 | M | 52 | AcoA, MCA | V |

ACA: anterior cerebral artery, PcoA：posterior communicating artery, AcoA: anterior communicating artery, MCA: middle cerebral artery, PICA: posterior inferior cerebellar artery, VADA: vertebral artery dissection aneurysm.
