# Supplementary figures and images for: Inhibition of AIM2 inflammasome activation alleviates GSDMD-induced pyroptosis in early brain injury after subarachnoid haemorrhage
Source: Cell Death Dis. 2020 Jan 30;11(1):76. doi: 10.1038/s41419-020-2248-z (PMC6992766; doi:10.1038/s41419-020-2248-z)

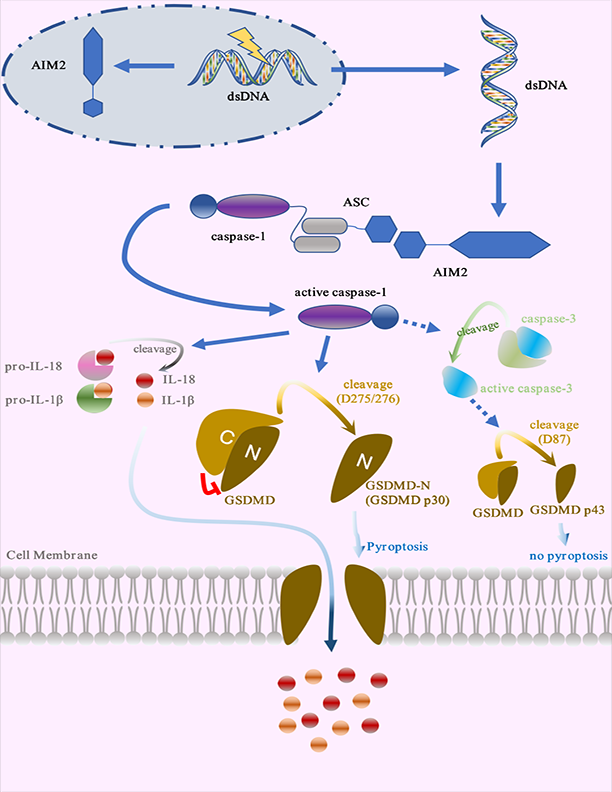

Supplement: Supplementary file 2 — Supplementary figure [file 41419_2020_2248_MOESM2_ESM.tif]
